# College students’ loneliness and mobile phone addiction: mediating role of mobile phone anthropomorphism and moderating role of interpersonal relationship quality

**DOI:** 10.3389/fpsyt.2025.1581421

**Published:** 2025-06-09

**Authors:** Huan Wang, Mingxin Ji, Huixin Tu, Yi Qi, Siyi Wu, Xingrui Wang

**Affiliations:** School of Economics and Management (School of Accounting), Yunnan Minzu University, Kunming, China

**Keywords:** loneliness, college students, mobile phone addiction, mobile phone anthropomorphism, interpersonal relationship quality

## Abstract

**Introduction:**

As mobile phone functions expand and diversify, users develop deep emotional bonds with their devices. This has led to a growing concern about mobile phone addiction, particularly among students in universities. Previous research on mobile phones dependency examined their functional impact on user addiction, with limited exploration of individual psychological mechanisms. Unlike prior research that primarily examines mobile phone addiction as a behavioral issue, this study introduces the concept of mobile phone anthropomorphism as a key psychological mechanism in addiction formation. This study examines the relationship between loneliness and phone addiction among students, focusing on the mediating role of phone personification and the moderating effect of relationship quality.

**Methods:**

In this research, the method of random cluster sampling was used to distribute network link questionnaires to universities across China. Descriptive statistical analysis and tests of mediation and moderation effects were conducted utilizing IBM^®^ SPSS^®^ 27.0, and PROCESS 4.1, respectively. A single-factor Harman’s test was used to assess common method variance, and Pearson’s correlation analysis was employed to explore the relationships between the major variables.

**Results:**

(1) A remarkable positive association existed between loneliness and smartphone dependency among students. (2) Mobile phone anthropomorphism functions as an intermediary in the link between loneliness and smartphone dependency among students. (3) Interpersonal relationships quality negatively regulate loneliness, mobile phone anthropomorphism, and smartphone dependency.

**Discussion:**

This study offers a fresh perspective on comprehending the psychological characteristics and mobile phone dependence of young people. Loneliness directly contributes to mobile phone addiction and indirectly reinforces dependence through anthropomorphism.When loneliness increases, college students rely more on mobile phones for emotional sustenance and increase unrealistic interactions, thereby facilitating the development of mobile phone addiction. Strong interpersonal relationships mitigate loneliness, reducing both mobile phone anthropomorphism and addiction risk.

## 1 Introduction

As the master of the information age, smartphones have pushed this extension to unprecedented heights. Regardless of whether it is communication interaction, daily travel, or office and leisure activities, smartphones have penetrated people’s daily lives and brought convenience to the public. However, empirical data reveals that approximately 285 million Generation Z youth worldwide exhibit tendencies toward excessive smartphone usage, with Chinese university students similarly confronting pronounced mobile social media addiction issues (1). This emergent phenomenon of smartphone addiction has ignited intensive scholarly investigations into the underlying mechanisms of “problematic mobile phone use”. Within the context of digital existence, interactive paradigms between young individuals and intelligent terminals have transcended conventional instrumental usage frameworks, progressively constructing quasisocial attachment relationships. This relational configuration not only manifests as behavioral-level functional dependence but also profoundly intervenes in the individual’s self-construction process through neurocognitive mechanisms, triggering categorical restructuring of self-identity and value system reconfiguration (2). Some young people report feeling as if their mobile phones act as extensions of their minds, allowing them to navigate seamlessly between the virtual and real worlds. This perception reinforces the role of mobile phones as a bridge between their internal and social selves (3). With the growing prevalence of personalized modifications to both the appearance and functionality of mobile phones, young adults are increasingly viewing these devices as essential tools for self-expression. This trend not only caters to users’ aspirations for uniqueness and self-identity but also fosters a more intimate and collaborative relationship between humans and their mobile devices (3, 4). Mobile phones have evolved from mere auxiliary tools into “intelligent companions” capable of deep interaction and understanding of user needs. As people perceive mobile phones as partners with human-like attributes, they are more likely to develop a sense of dependence and emotional attachment towards them. This emotional bond not only fulfills social needs but also provides psychological comfort and companionship (4). In the fast-paced and high-pressure environment of modern life, mobile phones, as extensions of one’s identity values, and emotional requirements, naturally serve as significant carriers of anthropomorphism, offering a unique means for individuals to mitigate loneliness and bolster psychological resilience (5).

Studies have shown that the experience of loneliness peaks at the age of 20 and is widespread across cultures, becoming an epidemic in modern society (6). Individuals with a strong sense of loneliness often face obstacles in social interactions in real life: however, despite such difficulties, they still have a need for social communication and a desire to obtain interpersonal satisfaction and connection (7). Attachment theory states that humans are born with the instinct to seek emotional attachment and that this attachment is not limited to human beings: people can also develop emotions similar to social attachment to nonhuman objects such as mobile phones. When mobile phones are unavailable, people experience physical and psychological stress (8). This phenomenon is particularly pronounced among lonely people who view their phones as amplifications of their thoughts and selves, and their phones are increasingly symbiotic with them (9). The original intention of people using mobile phones is to seek companionship and escape loneliness, and they expect to obtain interpersonal warmth and care through the mobile phone network space. However, the resulting problematic use of mobile social media as well as its negative impact on adolescents’ physical and mental development has become a major concern for society (10). The reality is that teenagers are gradually becoming immersed in the mire of the Internet (11). Technology has such a powerful appeal precisely because it touches the most vulnerable parts of people’s hearts. People are afraid of loneliness but also desire intimacy, so they use technology to build an intimate digital environment in which to enjoy virtual care and company (12). Both social penetration theory and anthropomorphic three-factor theory support the idea that anthropomorphism is a key step in establishing a relationship during initial human-computer contact (13, 14). Numerous studies have focused on various special smartphone applications to explore their impact on social adaptation and mental health, such as addiction to short videos, online shopping apps, and mobile games (15–17). However, most of these studies were limited to the functional level of mobile phones as objects, and exploration of the internal psychological mechanisms of addiction is relatively scarce. As a new and relatively unexplored research field, research on anthropomorphism in mobile phone addiction is particularly scarce.

While previous research has examined mobile phone addiction as a behavioral issue linked to app usage and screen time, the role of anthropomorphism-where users psychologically perceive phones as human-like partners has been largely overlooked. This study selected university students-a specific cohort experiencing a relatively elevated sense of loneliness-to explore why college students tend to regard mobile phones as human partners and have a strong dependence on them, and what is the connection between loneliness, mobile phone anthropomorphism, and phone addiction. How does the nature of the relationship, which is closely related to loneliness, affect functional relationships? This research revealed the complex mechanisms of loneliness, mobile phone anthropomorphism, and dependency on mobile phones. Understanding this phenomenon could offer novel insights into how mobile dependency develops and provide new intervention strategies.

## 2 Research hypotheses

### 2.1 Loneliness and mobile phone addiction among college students

Social need theory, cognitive difference theory, and the evolutionary perspective constitute the three theoretical pillars of the formation mechanism of loneliness. Social needs theory stresses that individuals’ loneliness is due to a lack of social relations, and people are born with the demand for six basic relationships, when these basic needs are not met, individuals will experience attacks of loneliness (18). Cognitive difference theory further explains the mechanism of loneliness from the perspective of individual subjective perception and points out that individuals’ sense of lack of basic relationship satisfaction is based on their cognitive differences. Loneliness emerges when a substantial disparity arises between the level of closeness individuals envision and the existing reality, resulting in the onset of loneliness (19).

Therefore, this paper posits that loneliness refers to the need for individuals to establish highquality relationships with their social members. When individuals perceive a deviation or inconsistency between the quality or quantity of expected social relationships and the actual situation, it may lead to unhappy subjective psychological feelings and avoidant communication behaviors (20, 21). Loneliness manifests as two ideologies: emotional and social loneliness. The former is due to the alienation of interpersonal relationships (intimate relationships), whereas the latter is due to a lack of social interaction and a sense of value (22). It frequently impacts both an individual’s physical (chronic disease and sleep quality) and psychological well-being (depression, anxiety, and psychotic symptoms) (23). The post-2000s generation is the new generation in the 21st century that has grown synchronically with the Internet and various advanced technologies. They are the “natives” of the information age, the beneficiaries and promoters of the Internet. However, in recent years, studies have shown that college students’ loneliness has gradually intensified over time (24) and an increasing number of college students use mobile phones to seek emotional comfort as a way to escape reality. In addition, college students who experience loneliness are more prone to smartphone addiction (21). As a core mediating variable, loneliness exacerbates addiction symptoms by weakening real-life social motivation and strengthening virtual compensatory dependence. When college students perceive inadequate social support, loneliness further drives them to seek instant emotional feedback through mobile games and social apps (25), forming a vicious cycle of “loneliness-virtual socializationaddiction reinforcement”. According to the induction susceptibility model, once factors related to mobile phones or mobile phone use are combined with incentives, an individual’s excessive and compulsive reliance on mobile phones escalates, resulting in smartphone addiction (26). It has been argued that loneliness has become one of the main factors inducing smartphone addiction in college students, and students who feel lonely are more prone to fall into the dilemma of smartphone addiction (22). This can be attributed to emotional elements that significantly influence interpersonal communication and affect individual perceptual mechanisms. When individuals suffer from isolation or external marginalization, their emotional state is easily damaged, and they automatically reject social activities conducive to physical and mental health (27) and instead tend to use mobile phones as a medium for information and emotional communication (25).

Therefore, this study proposes the following research hypothesis:

H1: Loneliness among college students may have a positive effect on mobile phone addiction.

### 2.2 Mediating effect of mobile phone anthropomorphism

Anthropomorphism comes from the combination of the Greek words “Anthropos” and “morphe”. It refers to the physical characteristics or ideologies that humans subjectively and deliberately assign to nonhuman objects. The core of anthropomorphism is not to create or observe the appearance of nonhuman objects but to believe that nonhuman objects have human mental and cognitive systems, including consciousness, emotions, and higher-order cognitive abilities (28). The theory of the three personification factors explains the reasons for their generation. Personification is believed to be mainly a psychological process based on cognitive and motivational factors. It also proposes the elicited agent knowledge, drive for effectance, and motivation for social connection, namely the SEEK models (16). Elicited agent knowledge means that when an individual perceives the human-like features displayed by an object, they will use the previously acquired human experience schema to explain their behavior-that is, to themself-and they will transfer the general knowledge in human life to the object. Effectance motivation means that when an individual is in a strange and uncertain environment, it produces emotional experiences such as panic and anxiety. To become familiar with their surrounding environment and reduce negative emotions, individuals personify nonhuman entities.When an individual’s social needs remain unfulfilled, people tend to anthropomorphize non-human objects to make up for the individual’s sense of social belonging and companionship (29, 30). As mobile phones have become increasingly intelligent, many people regard them as loyal and intimate partners, generating a strong sense of trust and happiness when they get along with them (2). The “human touch” of mobile phones is born from the ability of human beings to subjectively imagine that mobile phones have positive emotional feedback. Human beings psychologically empower mobile phones, believing that they have personality traits, emotions, and the ability to perceive the world, have a strong emotional connection with themselves, and even incorporate them into themselves (31). When individuals strive to establish high-quality relationships with members of society and perceive a lack in either the quality or quantity of the anticipated social ties, they may experience feelings of loneliness. This loneliness not only has the potential to directly propel mobile phone addiction behavior but may also indirectly exacerbate such addiction by prompting individuals to perceive their mobile phones as emotional support entities, thereby anthropomorphizing them.

College students are semi-detached from their families and face an unfamiliar environment at school alone, which produces an urgent need to establish deep emotions to familiarize themselves with the environment. If the establishment is frustrated, it can lead to loneliness and mental illness (23). However, if an individual continues to be lonely, this part of the population may abandon conventional social willingness and the tendency to establish interpersonal relationships and instead tend to seek emotional connections with nonhuman objects through anthropomorphic methods (32). For people with high loneliness, anthropomorphic objects are often regarded as more reliable and easier to build stable connections with than human beings. These objects replace the role of interpersonal communication and become an important source of security and comfort for individuals (33). The social cognitive theory suggests that humans judge the degree of anthropomorphism of objects based on warmth (caring, friendliness, and kindness) and efficacy. The higher the degree of anthropomorphism perceived by the object, the higher the degree of emotional involvement would be (34). Today, the relationship between young people and mobile phone is becoming increasingly intimate (35). When lonely people perceive the warmth of mobile phones and seek psychological comfort, they are often more inclined to regard mobile phones as partners with whom they can communicate and rely on (36). A positive association, may exist between anthropomorphism and mobile phone addiction (37), and mobile phone self-inclusion plays a complete mediating role between mobile phone anthropomorphism and mobile phone self-expansion (31), while mobile phone self-expansion can positively predict smartphone addiction (38). The cognitive behavioral model points out that an individual’s cognitive activities directly determine his emotional and behavioral responses rather than the external environment, and the key lies in how an individual interprets external stimuli (39). At the cognitive level, college students who experience high levels of loneliness may be more prone to cognitive bias and false beliefs that mobile phone use is an effective way to relieve stress, escape from reality, or find fun and thus project excessive emotional dependence on mobile phones (6). In terms of emotions, mobile phone personification can meet the needs of lonely university students by making them feel understood, accompanied, or concerned. In terms of behavior, lonely students may form specific behavioral habits in the process of interacting with mobile phones (1), and these behavioral habits may be strengthened by the anthropomorphic influence of mobile phones. Over time, an individual may gradually formed a kind of mobile phone addiction behavior patterns.

Consequently, this study puts forward the following research hypothesis:

H2: Anthropomorphism in mobile phones may play a mediating role between loneliness and mobile phone addiction.

### 2.3 Moderating influence of interpersonal relationship quality

The quality of social connections refers to a network of mutual influence and communication among all members of society, which plays a significant role in fulfilling an individual’s needs. This study considers interpersonal relationship quality broadly, including relationships with friends, colleagues, partners and family. It reflects the depth of mutual understanding, trust, dependence, and influence between individuals. The closeness of an intimate relationship between individuals has a significant impact on their behavioral patterns and decision-making processes. In close relationships, this kind of relationship is reflected in the tendency for cooperation. Conversely, interaction with strangers is more limited to simple task interaction, which leads to a reduction in social interaction and emotional exchange (40).

For college students who spend at least 60% of their time in a dormitory, dormitory relationships are a key indicator of the quality of their interpersonal relationships. However, the quality of dormitory relationships showed an annual downward trend during the four years of this study (41). If an individual is in an environment with poor-quality interpersonal relationships for a extended period, It is simple to close the door on emotions, resulting in feelings of loneliness (42) and increased fear of revealing their true self. Therefore, loneliness is primarily caused by interpersonal defects (33). According to the interpersonal relationship theory, if an individual feels lonely in interpersonal communication, it will not only lead to depression and social withdrawal behavior but also affect the individual’s subsequent behavior pattern (43), prompting the generation of anthropomorphic behavior of the mobile phone. Driven by loneliness, individuals may be more inclined to regard mobile phones as objects of emotional sustenance and seek comfort and satisfaction through frequent interactions with mobile phones (44). Among university students who experience feelings of loneliness, this behavior of personifying mobile phones compensates the emotional communication that individuals do not get in real life, enhances their vacant sense of social connection, and can alleviate individual loneliness to a certain extent. Specifically, In the course of interpersonal interaction among students, people who present a lower level of interpersonal relationship quality are more likely to strengthen their sensations of solitude and thus tend to anthropomorphize mobile phones. Among the factors of loneliness, mobile phone personification, and mobile phone addiction, the quality of interpersonal relationships plays a negative moderating role.

The greater the loneliness of individuals, the more robust their psychological defense mechanisms and the more difficult it is to develop high-quality interpersonal relationships in real life. Owing to the instantaneity and diversity of virtual social networks, offline social support resources such as information acquisition, self-esteem satisfaction, emotional communication, and peer support can also be satisfied in the online virtual space (27). College students experiencing significant loneliness often leverage the social features of the Internet to establish a broad range of online social networks. which can carry out instant online communication with classmates, friends and virtual netizens anytime and anywhere, but this often leads to overreliance on smartphones, which leads to obsessive actions (45). Communication between individuals occurs in the interpersonal relationship network established by individuals.Family, friendships, and love play a pivotal role. High-quality interpersonal relationships are conducive to the healthy development of individuals and have a positive effect on interactions between members and mutual connections between individuals and groups (40). Empirical studies suggest that in high interpersonal interactions, university students can establish positive self-identity and enhance their self-confidence and self-esteem through active engagement with others. This positive psychological state renders them more resilient to the temptation of phone anthropomorphism and addiction (45). According to the resilience framework theory, various types of support, as key components of resilience, play a regulatory and protective role in the relationship between risk factors and personal development, effectively minimizing the adverse effects caused by risk factors, thus promoting individual psychological adjustment and overall development (46). For university students, these forms of support not only offer emotional solace, effectively mitigating the adverse impacts of various risk factors, but play a pivotal role in fostering positive self identification and a profound sense of belonging. As the perception of subjective interpersonal support intensifies in their real-life experiences, their internal senses of belonging and identity undergo a corresponding enhancement. When their emotional requirements are adequately met within the real world, the impetus to excessively seek solace within the realm of virtual social interactions diminishes, thereby significantly decreasing their reliance on mobile phones (45).

This study therefore proposes the subsequent hypothesis:

H3: The quality of interpersonal relationships serves as a negative moderator in the relationships among loneliness, mobile phone anthropomorphism, and mobile phone addiction.

**Figure 1:**
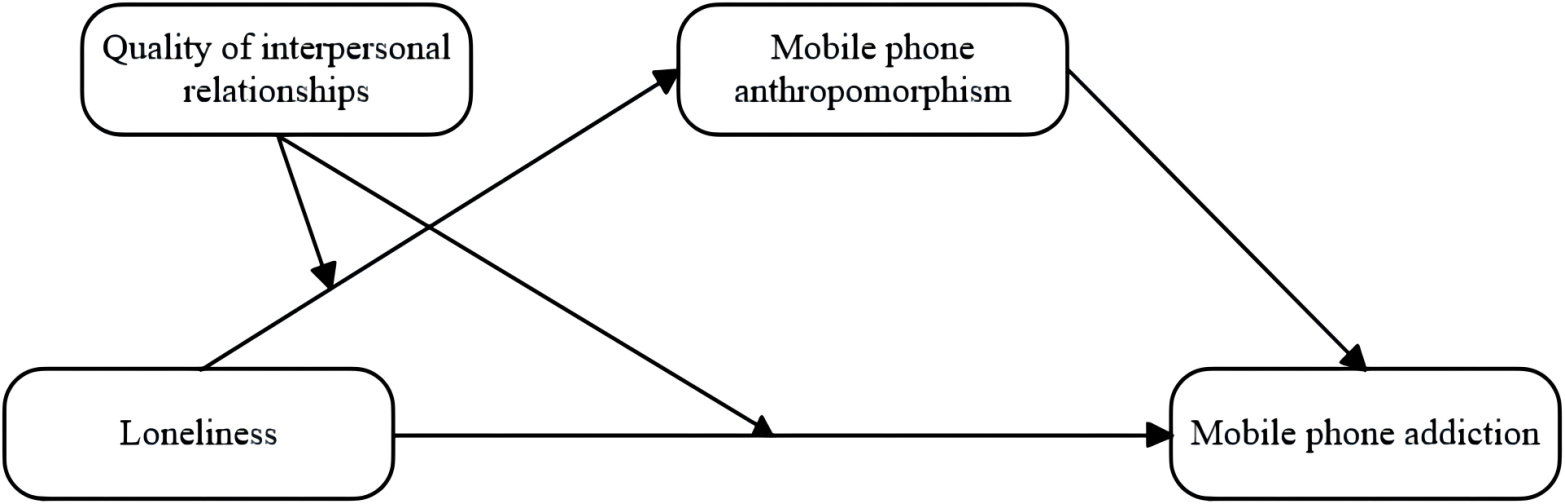
Mediating model of loneliness and mobile phone addiction among college students.

To sum up, in this study, the mediating regulation model illustrating the connection between loneliness and mobile phone addiction among college students is presented in **Figure 1**.

## 3 Methods

### 3.1 Sample and process

The study adopted a random cluster sampling method and questionnaires were sent to students of ordinary colleges and universities across China in the form of online questionnaire linksfrom November 26, 2024 to December 4, 2024. The study targeted full-time undergraduate

students from regular 4-year universities in China. A “regular university” in the Chinese higher education system refers to government-accredited institutions offering bachelor’s degree pr-ograms, typically with on-campus dormitories and a structured curriculum.In China, most undergraduate students live in on-campus dormitories, regardless of geographic proximity to the-ir hometowns. This uniform residential context reduces variability in living arrangements. In

total, 1,501 valid questionnaires were obtained. The participants were distributed across 29

provinces, municipalities or autonomous regions in China. Among them, the provinces with arelatively large proportion were Guangdong province with 169 participants, accounting for 11.26%. Heilongjiang province with 154 participants, accounting for 10.26%. Jiangsu province

with 120 participants, accounting for 7.99%. Shandong province with 119 participants, accou-nting for 7.93%. Henan province with 117 participants, accounting for 7.79%. Zhejiang prov-ince with 104 participants, accounting for 6.93%; and Hebei province with 85 participants,

accounting for 5.66%. The age of the participants varied between 18 and 23 years old, with158 aged 18 years (10.53%), 400 aged 19 years (26.65%), 377 aged 20 years (25.1 2%), 367 aged 21 years (24.45%), 167 aged 22 years (11.13%), and 32 aged 23 years (2.13%).

Among them, 700 were boys (47%), 801 were girls (53%). There were 364 first-year studen-ts (24.25%), 382 sophomores (24.45%), 390 junior-year students (25.98%), and 365 senior-ye-ar students (24.32%). All study participants are all full-time undergraduate students from regular universities across the country (excluding international students).

### 3.2 Questionnaire design

#### 3.2.1 Mobile phone addiction tendency scale for college students

The survey assessing college students’ tendencies towards mobile phone addiction utilized the scale measuring their addiction propensity to mobile phones (47). The measurement instrument is centered on exploring university students’ smartphone usage patterns and addictive tendencies, possessing a high level of comprehensiveness and investigative depth. The questionnaire consisted of 16 questions distributed across four dimensions: withdrawal symptoms, conspicuous behavior, social comfort, and emotional changes. Specifically, these dimensions included 6 items for withdrawal symptoms, 4 items for notable behavior, 3 items for social comfort, and 3 items for emotional changes. For evaluating tendencies toward smartphone dependency among university students, a five-point scoring system was used in the questionnaire, with higher scores reflecting a stronger inclination toward phone addiction. The Cronbach’s alpha coefficient for this scale was 0.893. The content of the scale is shown in **Table 1**.

**Table 1:** Aspects and themes covered by the scale assessing smartphone dependency tendencies among university students.

Scale name	Scale dimension	Title
Mobile phone addiction tendency scale for college students	Withdrawal symptom	For a period of time without phone, I’ll go to check whether there is a message/call.
		I feel bad if I don’t use my phone for a long time.
		If there is no mobile phone I will feel lonely.
		When my phone doesn’t ring for a while, I feel uncomfortable and subconsciously check my phone for missed calls/texts.
		More phone calls and more text messages make my life more fulfilling.
		My phone is a part of me, and when I reduce it, I feel like I’m losing something.
	Conspicuous behavior	I can’t pay attention in class because of phone calls or text messages.
		I often have the illusion of “my phone is ringing/my phone is vibrating”.
		My classmates and friends often say that I rely too much on my mobile phone.
		In class, I often take the initiative to focus on my mobile phone and affect my listening.
	Social comfort	I’d rather chat on my phone than have a face-to-face conversation.
		I feel more confident when I use my phone to communicate with others.
		I feel more comfortable communicating with others by mobile phone.
	Mood change	While waiting for someone, I often call them on my mobile phone to ask where they are and get anxious if I don’t.
		I often fear that my phone will turn off automatically.
		When my phone often doesn’t connect or get a signal, I get anxious and my temper gets cranky.

#### 3.2.2 Loneliness scale

The scale for measuring loneliness (48), originally devised by Russell and later modified by Wang, was employed. The scale encompasses 18 items scored on a 4-point Likert scale, varying from 1 (representing strong disagreement) to 4 (representing strong agreement), among which items 1, 4, 5, 6, 8, 9, 14, 15, 16, and 18 were scored in reverse. Scores that are higher reflect increased levels of lonesomeness. The internal consistency of this loneliness assessment, as indicated by Cronbach’s alpha, was 0.968. The items comprising the scale are detailed in **Table 2**.

**Table 2:** Questions of loneliness scale.

Scale name	Title
Loneliness scale	I feel I get along well with the people around me.
	I feel that I lack the friendship of others.
	There was no one around me to whom I could turn for help.
	I don’t feel lonely.
	I feel I am a member of the companions.
	I think I have a lot in common with the people around me.
	My interests cannot be shared by those around me.
	I am an easy person to get along with people.
	There are people around that I feel close to.
	I feel forgotten by others.
	I feel disconnected from the people around me.
	I felt that no one really understood me.
	I feel so isolated.
	I have someone to be with when I need them.
	I can find someone to talk to.
	There are people around me whom I can turn to for help.
	I feel that I lack a common language with the people around me.
	I feel like someone cares about me.

#### 3.2.3 Mobile phone anthropomorphic scale

A mobile phone anthropomorphism scale (49) was used to measure the degree of anthropomorphism of the subjects. There were 14 items, including two dimensions (1): direct reflection of anthropomorphism, such as “my mobile phone has its own intention” (2). the perception of mobile phone anthropomorphic (indirectly reflect), such as “the mobile phone has the ability to infringement of personal rights and freedoms.” The items were rated using a 7-point scale, with 1 representing strong disagreement and 7 representing strong agreement. The greater the score, the more anthropomorphic the phone appears. In this research, the Cronbach’s alpha coefficient of anthropomorphic mobile phone was 0.962. The specifics of the scale are outlined in **Table 3**.

**Table 3:** Dimensions and topics of mobile phone anthropomorphic scale.

Scale name	Scale dimension	Title
Mobile phone anthropomorphic scale	The direct reflection of anthropomorphic	My phone is powerful
		My phone is very attractive.
		My phone is very efficient.
		My phone has a lot of personality.
		My phone is conscious.
		My phone has its own mood.
		My phone has a mind of its own.
		My phone can sense emotions.
		My phone has its own purpose.
	Anthropomorphic perception of mobile phone (indirect reflection)	When I play a game with my cellphone, I worry it might cheat.
		I used a phone that didn’t like me.
		My phone can control my movements.
		My phone is an invasion of my privacy.
		My phone may violate individual rights and freedoms.

#### 3.2.4 General interpersonal goals scale

The general interpersonal goals scale, developed by Canevello and Crocker and later refined by Min Zhang, Lin Zhang, and Corcke, was utilized to assess interpersonal relationships (50). The questionnaire comprises 18 questions, each scored on a 5-point Likert scale ranging from 1 (Intensely inconsistent) to 5 (Intensely consistent). Increased scores suggest enhanced human relationships. In this research, the Cronbach’s alpha reliability coefficient for the general interpersonal communication goal scale was 0.967. **Table 4** presents the constituent components of the scale.

**Table 4:** Dimensions and topics of the general interpersonal communication goal scale.

Scale name	Ttitle
General interpersonal goals scale	Avoid being selfish or self-centered.
	Accept the mistakes or shortcomings of others.
	Avoid focusing on your own needs without considering the needs of others.
	Understand the impact of your actions on others.
	Support and encourage others.
	Avoid doing things that are not beneficial to yourself or others.
	Do something that helps you or someone else.
	Avoid doing things that will hurt others.
	Avoid neglecting your relationships with others.
	Make others know or recognize your intelligence.
	Draw attention to your positive qualities.
	Show others what you do best.
	Avoid coming across as ignorant, incompetent, or unintelligent.
	Avoid revealing your weaknesses.
	Convince others that you are right.
	Do something you think will succeed.
	Act helpful.
	Avoid being criticized or blamed by others.

### 3.3 Data processing and statistical analysis

IBM^®^ SPSS^®^ 27.0, and Process 4.1, were utilized to conduct descriptive statistical analysis and model testing of mediating and moderating effects, respectively. To assess potential common method variance, we employed the Harman’s one-factor test. Furthermore, We conducted a Pearson correlation analysis to explore the associations between the primary variables.

### 3.4 Common method bias test

In this research, specific control measures were implemented, including anonymous surveys and the use of reversed scoring for certain questions within the program. Meanwhile, in order to assess common method variance, we utilized the Harman one-factor test. The analysis showed that seven factors had eigenvalues exceeding the threshold of one, with the dominant factor accounting for 35, 95% of the variance, falling below the crucial cut-off point of 40%. Consequently, this research failed to identify a significant common method bias problem.

## 4 Results

### 4.1 Descriptive statistical analysis

The associations among variables were assessed using Pearson’s correlation coefficient (**Table 5**). There was a significant positive correlation between mobile phone addiction and loneliness (*r*=0.56, *p*<0.01). Our findings indicate a moderate relationship, reinforcing previous research while highlighting the particular vulnerability of college students. Thus, H1 was verified. There was a significant positive association between mobile phone addiction and anthropomorphism (*r*=0.58, *p*<0.01). There was a moderate degree of positive correlation between the two variables, illustrating the mechanism of action of the second path variable of the mediation process. There existed a notable negative correlation between mobile phone addiction and interpersonal relationships *(r*=-0.57, *p*<0.01). It shows that there is a moderate relationship between the two, and interpersonal relationship quality has a certain degree of influence on mobile phone addiction behavior. Loneliness exhibited a positive correlation with mobile phone anthropomorphism (*r*=0.43, *p*<0.01), and negatively correlated with interpersonal relationship (*r*=-0.40, *p*<0.01). Mobile phone anthropomorphism was significantly negatively correlated with interpersonal relationships(*r*=-0.44, *p*<0.01). The study reveals a moderate association among loneliness, mobile phone anthropomorphism and interpersonal relationship. Additionally, the correlation between gender, grade, and the study variables was not significant, therefore, no control variables were included in the subsequent analysis.

**Table 5:** Standard deviation, mean value and correlation matrix of each variable (N=1501).

	M	SD	Gender	Grade	Mobile phone addiction	Loneliness	Mobile phone anthropomorphism	Interpersonal relationship
Gender	1.53	0.50	1.00					
Grade	2.50	1.11	0.01	1.00				
Mobile phone addiction	3.37	0.89	-0.01	-0.02	1.00			
Loneliness	3.36	1.16	0.01	-0.03	0.56**	1.00		
Mobile phone anthropomorphism	4.53	1.37	0.02	-0.02	0.58**	0.43**	1.00	
Interpersonal relationship	2.59	1.14	-0.09	0.03	-0.57**	-0.40**	-0.44**	1.00

**p<0.01.

### 4.2 Loneliness and mobile phone addiction: a moderation mediation model test

#### 4.2.1 The mediating effect analysis of mobile phone anthropomorphism

Model 4 in the SPSS macro program PROCESS, prepared by Hayes (Model 4 for simple mediation model), was used to inspect the mediation role of mobile phone anthropomorphism between loneliness and mobile phone addiction. The results are shown in **Tables 6** and **7**. Loneliness has a significant predictive effect on mobile phone addiction (*β*=0.43, *t*=26.07, *p*<0.001), loneliness of direct prediction effect of mobile phone addiction was still significant after the intermediary variable of interpersonal relationships was introduced (*β*=0.30, *t*=17.85, *p*<0.001). Loneliness predicted mobile phone anthropomorphism significantly (*β*=0.51, *t*=18.65, *p*<0.001), and mobile phone anthropomorphism predicted mobile phone addiction significantly (*β*=0.27, *t*=19.32, *p*<0.001). In addition, the mediation effect was tested using the deviation corrected percentile bootstrap method with 5000 sampling times. The 95% bootstrap confidence interval for the direct impact of loneliness on mobile phone addiction, as well as the intermediary role of mobile phone anthropomorphism, did not encompass the value of 0 for both its lower and upper bounds. as shown in **Table 7**, which indicates loneliness can not only serve as a direct predictor of mobile phone addiction, but also positively influences it through the mediation of mobile phone anthropomorphism. Thus, H2 was verified. The direct (0.29) and intermediate (0.14) effects accounted for 67.44% and 32.56% of total effect (0.43), separately.

**Table 6:** Mediating effect test of mobile phone anthropomorphism.

Regression equation(N=1501)		Fitting index			Significance coefficient	
Result variable	Predictor	R	R²	F	β	T
Mobile phone addiction		0.56	0.31	679.46***		
	Loneliness				0.43	26.07***
Mobile phone anthropomorphism		0.43	0.19	347.83***		
	Loneliness				0.51	18.65***
Mobile phone addiction		0.67	0.45	610.71***		
	Loneliness				0.30	17.85***
	Mobile phone anthropomorphism				0.27	19.32***

***P < 0.001.

**Table 7:** Decomposition diagram of total effect, direct effect and mediation effect.

	Effect value	Boot standard error	Boot CI lower limit	Boot CI upper limit	Relative effect size
Total effect	0.43	0.02	0.49	0.46	
Direct effect	0.29	0.02	0.26	0.32	67.44%
Mediation effect	0.14	0.01	0.12	0.16	32.56%

#### 4.2.2 The moderating effect analysis of interpersonal relationship

Model 8 in the SPSS macro program PROCESS compiled by Hayes (Model 8 posits adjustments to both the initial segment of the mediation process and the direct pathway, aligning with the theoretical framework used in the research) was used to inspect the mediation model with adjustment. The results are presented in **Tables 8** and **9**. After adding interpersonal relationships into this model, the interaction between loneliness and interpersonal relationships significantly predicted a decrease in mobile phone addiction and anthropomorphism (mobile phone addiction: *β*=-0.16, *t*=12.32, *p*<0.001, mobile phone anthropomorphism: *β*=-0.27, *t*=-11.27, *p*<0.001), indicating that interpersonal relationships not only negatively moderated loneliness’ direct prediction on mobile phone addiction but also negatively moderated the prediction of loneliness on mobile phone anthropomorphism. Thus, H3 was verified. It can be said that among people with loneliness, the influence degree of interpersonal relationship on mobile phone addiction is -0.16, for example, in the environment of high-quality interpersonal relationship, the dependence degree of lonely people on mobile phones will be relatively reduced by 16%.

**Table 8:** Tests for moderated mediating effects.

Regression equation(N=1501)		Fitting index			Significance coefficient	
Result variable	Predictor	R	R²	F	β	t
Mobile phone anthropomorphism		0.57	0.33	245.56***		
	Loneliness				0.34	12.66***
	interpersonal relationship				-0.35	-12.59***
	Loneliness×interpersonal relationship				-0.27	-11.27***
Mobile phone addiction		0.75	0.57	486.14***		
	Loneliness				0.24	15.78***
	interpersonal relationship				-0.24	-15.71***
	Mobile phone anthropomorphism				0.15	11.42***
	Loneliness×interpersonal relationship				-0.16	-12.32***

***P < 0.001.

**Table 9:** Direct effects and mediating effects at different levels of interpersonal relationship.

	Interpersonal relationship	Effect value	Boot standard error	Boot CI lower limit	Boot CI upper limit
Direct role	-1.14(M-SD)	0.42	0.02	0.38	0.46
	0(M)	0.24	0.02	0.21	0.27
	1.14(M+SD)	0.06	0.02	0.02	0.1
Mediating role	-1.14(M-SD)	0.1	0.01	0.08	0.12
	0(M)	0.05	0.01	0.04	0.07
	1.14(M+SD)	0.05	0.01	-0.01	0.02

**Figure 2:**
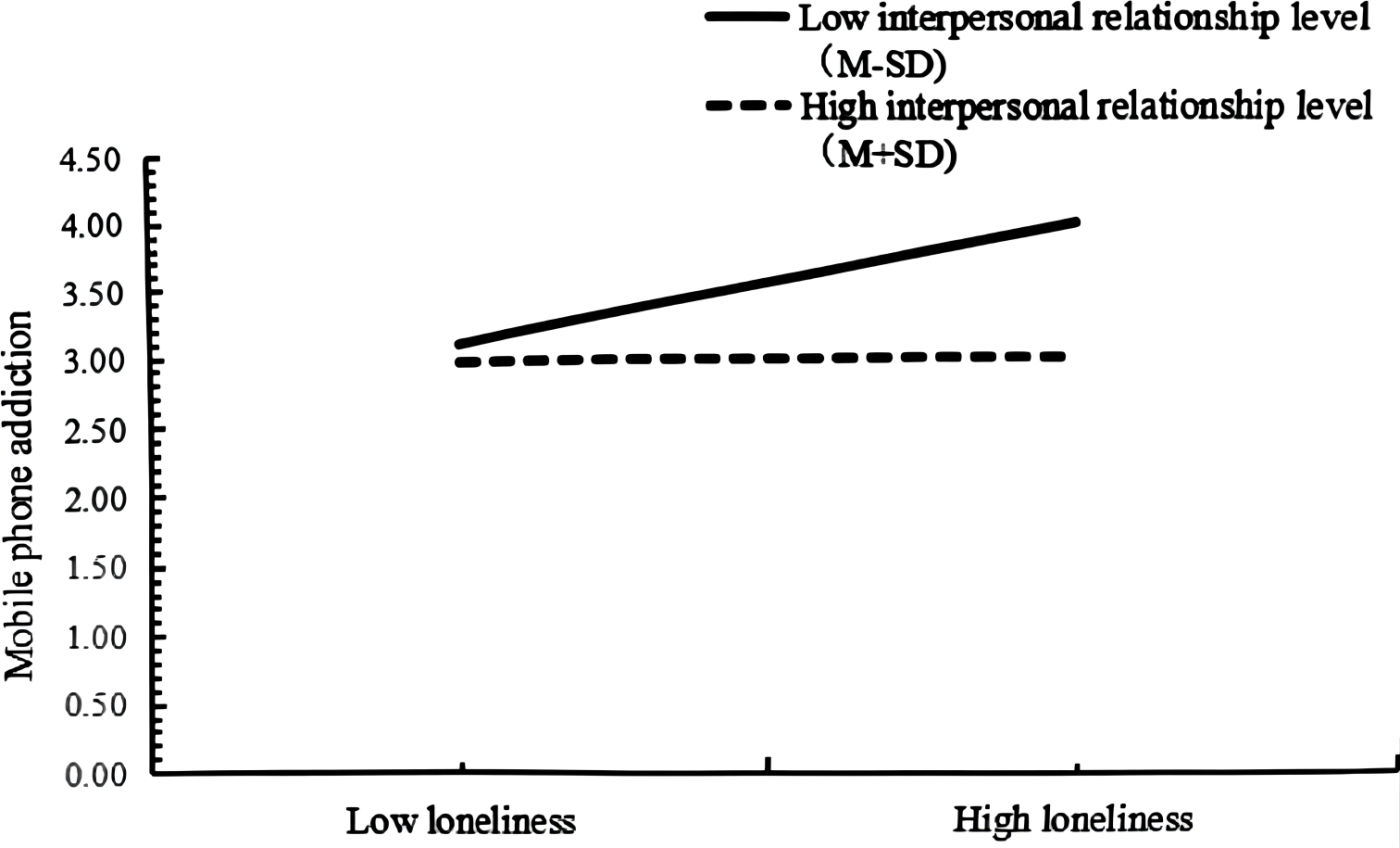
The moderating role of interpersonal relationship in the relationship between loneliness and mobile phone addiction.

**Figure 3:**
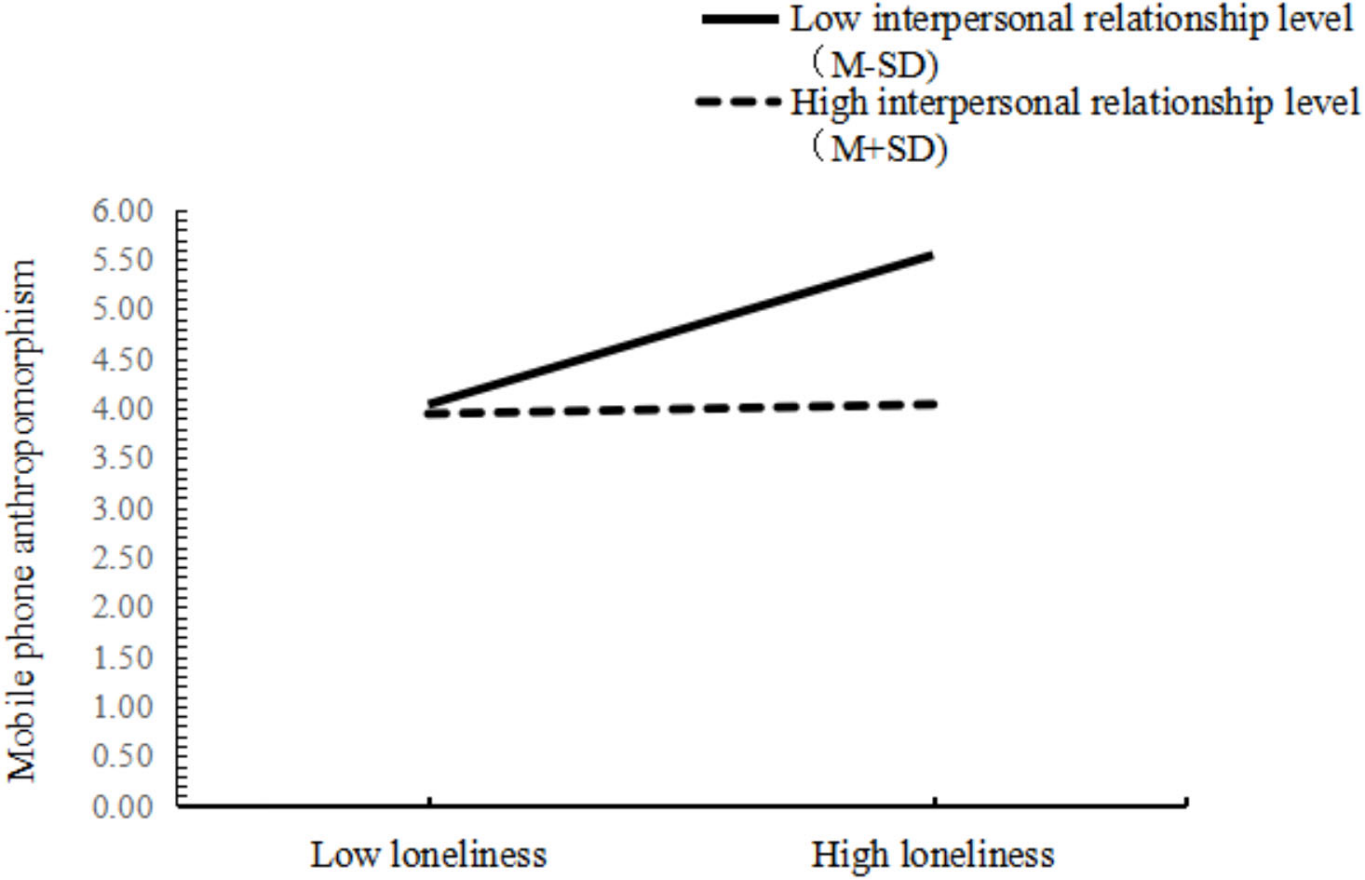
The moderating role of interpersonal relationship in the relationship between loneliness and mobile phone anthropomorphism.

To more intuitively observe the moderating role of interpersonal relationship in the direct pathway and the second half pathway of mediation model, the data were categorized into high groups and low groups (on the basis of ± *SD*) for simple slope analysis, as shown in **Figures 2** and **3**. As shown in **Figure 2**, for participants of low interpersonal relationship levels *(M-SD*), the loneliness had a remarkable positive predictive function on mobile phone addiction (*β*=0.42, *t*=19.56, *p*<0.001). For subjects with high level of interpersonal relationship levels (*M* ± *SD*), although loneliness had a remarkable predictive function on mobile phone addiction, its predictive function was small (*β*=0.06, *t*=2.70, *p*<0.01), instructing that with the augmentation of interpersonal relationship level, loneliness was a diminishing predictor of phone addiction, as shown in **Table 9**. According to **Figure 3**, for participants with low level of interpersonal relationships (*M-SD*), loneliness had a remarkable positive predictive effect on mobile phone anthropomorphism (*β*=0.65, *t*=17.44, *p*<0.001). For participants with high interpersonal levels, loneliness had no remarkable predictive effect on mobile phone ant hropomorphism (*β*=0.04, *t*=1.04, *p*>0.05). As illustrated in **Table 9**, at this three levels of interpersonal relationships, the mediating function of mobile phone anthropomorphism on the relationship between loneliness and mobile phone addiction also showed decreasing trend, that is, when the level of interpersonal relation of the subjects improved, loneliness was less likely to remit the degree of mobile phone addiction by reducing the degree of mobile phone anthropomorphism among college students.

## 5 Discussion

### 5.1 Loneliness is positively and significantly associated with mobile phone addiction

Loneliness significantly impacts mobile phone addiction, aligning with prior research (51–54). The evolutionary hypothesis of loneliness suggests that humans have evolved an instinctive tendency to maintain social bonds (55). Humans are born adaptable to their living groups. If they lack social and long-term interactions with others, they may suffer from loneliness and rejection. This experience can stimulate a deep inner driving force, prompting individuals to seek social contact to alleviate their loneliness. In contemporary society, mobile phones-a popular communication medium-have become the dominant mode of social contact. Based on media system dependence theory, people use media to achieve specific goals and become dependent on media because of its criticality or superiority in achieving these goals (56). Some scholars link excessive mobile phone use to int erpersonal indifference, trust erosion, and heightened social anxiety. At the same time, in virtual mobile phone communication, many people talk freely, but in real life, they are silent and lack of initiative, as if they lack communication ability in network communication (10). Overreliance on mobile phones for social interactions may weaken an individual’s interpersonal skills in the real world, thus inducing phone-dependent behaviors. The cognitive bias theoretical framework suggests that loneliness arises from a significant gap between an individual’s expectations of social participation and the reality of the situation, which can lead to significant loss of social networks or interpersonal dysfunction (24). In the process of exploring cyberspace to seek social belonging and personal vision realization, individuals tend to integrate reality with the virtual world, and this phenomenon of “borderless integration” encourages people to seek solace through the Internet to fill the lack of real life. When people get emotional support through online interaction, although they can gain a sense of belonging online, they may fall into the dilemma of social alienation in reality (45). Our findings align with Compensatory Internet Use Theory, as college students with high loneliness scores reported significantly higher mobile phone addiction. This supports the idea that mobile phones serve as an emotional escape mechanism, reinforcing dependence and addiction (57). As a medium of escapism, mobile phones can temporarily free individuals from difficulties and negative emotions. However, avoidance strategies may intensify dependence on mobile phones and lead to addiction (58). The self-regulation deficit model emphasizes that people with psychosocial problems and insufficient self-regulation and control may uncontrollably increase their mobile phone use, thus contributing to the maturation of mobile phone dependence (59), people who experience intense loneliness are often less self-controlled, more incline to find solace in digital realms, more likely to find it difficult to maintain healthy media habits, and more likely to become mobile phone dependent than individuals who experience less loneliness (60). Mobile phone addiction, mobile phone dependence, and loneliness are tightly connected. College students’ loneliness directly predicts mobile phone addiction severity and college students with strong self-perceived loneliness often show a stronger tendency toward mobile phone addiction and dependence (61, 62).

### 5.2 Mobile phone anthropomorphism plays a mediating effect between loneliness and mobile phone addiction

Mobile phone anthropomorphism mediates the loneliness-mobile phone addiction link. Loneliness fosters mobile phone addiction by increasing phone anthropomorphism. Regarding the first route of the mediation process, the paper found that people who have a strong sense of loneliness are more probable to personify their mobile phones. This is similar to scholars’ conclusion that long-term loneliness can strengthen people’s anthropomorphism toward nonhuman objects (32). Preference theory proposes that unpleasant experiences in interpersonal communication in a social environment cause people to change their propensity to pay attention, which leads to personal internalization problems and affects their behavioral presentation (63). People often participate in social and interpersonal communication to meet their emotional or material needs. Failure to meet these needs in social activities can lead to psychological problems in extreme cases, resulting in profound psychological experiences of loneliness. If one cannot talk to others about one’s negative emotions, it will be difficult to digest them. When individuals feel extremely lonely and misunderstand and distort their real social environment, they tend to use mobile phones to address their emotional needs (64). With the improvement of interactivity and intelligence, as well as their portability and versatility in providing instant feedback and interaction, mobile phone, in a way, simulate the experience of interpersonal communication and gradually play the role of human communication, becoming the emotional sustenance of people with high loneliness (65). According to media identity theory, people and media are in a social and natural communication and interaction relationship, and the small social clues sent by the media will arouse the natural social response of individuals, and they will interact with the media just as they communicate with real people, realizing the unconscious intersubjective communication (66). Huawei’s “Xiaoyi” Xiaomi’s “Xiao Ai” Apple’s “Siri” OPPO’s “Xiao Bu” each smartphone brand has its own voice assistant, which in addition to providing users with similar mobile phone guidance help such as checking the weather, setting alarms, or calling someone, but also can have emotional conversations. The factors influencing the virtual online experience of AI voice assistant users include perceived humanness, relationality, and perceived entertainment (67). The perception of quasihuman nature reduces users’ loneliness during the process of use, brings extreme emotional support to users, and even gives users an exclusive emotional relationship and perceptual experience with AI voice assistants (67). In this context, individuals with high grades of loneliness are more probable to anthropomorphize their mobile phones. Empirical research on the interactive experience of AI voice assistants was not involved in this study, which can be used as a research direction in the future.

Regarding the second way about the process of mediation, we discovered a remarkable positive connection between mobile phone anthropomorphism and addiction. Whether it is the concern about attachment to inanimate objects during isolation mentioned in the Western scriptures, or the importance of statues and idols in Western religion, it is clear that anthropomorphism is common. Anthropomorphism has both advantages and disadvantages, but this study is more inclined to the disadvantages of anthropomorphism (33). The negative effects of mobile phone anthropomorphism and mobile phone addiction in this paper are not self-evident, but are made clear by referring to relevant literature: the anthropomorphism of mobile phones may cause users to feel anxious and upset without their phones, thus exacerbating their addictive behaviors. In order to make users spend more energy and time, algorithm operators create an immersive human-computer interaction experience scene, which can meet people’s social needs, but also cause deep dependence, forming an “information cocoon”, solidifying thinking and narrowing vision (68). In recent years, ways to communicate with virtual characters through AI technology have become increasingly diverse, and relevant technologies have become increasingly mature. Through emotional dialogues and interactions with virtual characters, individuals acquire a sense of acquisition and experience emotional value. In one’s social life, positive spiritual power is sufficient to support individuals. Therefore, the anthropomorphic manner of talking to virtual characters may cause emotional dependence. According to attachment theory, users tend to devote more resources to objects to which they are emotionally attached (69). An emotional connection to mobile phones may lead to heavy mobile phone use behavior, causing individuals to spend more time alone with mobile phones, become immersed in their own world, and spend less time with human objects (49). Compared with smartphone functional dependence, smartphone identity is more likely for users to use their phones in depth (70). In other words, mobile phone belonging and anthropomorphism are more probable to induce mobile phone addiction. Studies have discovered that entities seek stronger stimuli because of weaker neural responses, which in turn lead to a need for more intense stimuli to satisfy the ego (71). In other words, after individuals personify their mobile phones for a long time, their interest in them decreases. To arouse the same degree of excitement, individuals strengthen their mobile phone anthropomorphism, which is more likely to cause mobile phone addiction. The process leads to an additional rise in mobile phone usage duration and finally, a vicious circle is formed. If the withdrawal mechanism of mobile phone dependence is lacking, psychological problems such as anxiety and depression will be more likely to occur, affecting the quality of sleep (68).

### 5.3 Moderating effect of interpersonal relationship quality on the relation between loneliness and mobile phone anthropomorphism and mobile phone addiction

This study found that the quality of interpersonal connection can adjust the influence of loneliness on mobile phone addiction: the greater the quality of relationships, the smaller the negative impact of loneliness on phone addiction. When the quality of interpersonal relationships is poor, loneliness has a significant negative impact on mobile phone addiction. The study design did not prove that mobile phone use caused a deterioration in relationship quality. The social reinforcement model explains the connection between access to social support and mobile phone dependence (72). Those who feel lonely because of the huge difference between reality and ideals or the disproportionate effort and return, if they have high-quality interpersonal relationships in real life, tend to have better emotional regulation ability than people with low-quality interpersonal relationships and tend to not turn to the network for depression. Therefore, the occurrence of mobile phone usage is reduced, and the grade of dependence on phones is reduced. The developmental loss compensation hypothesis holds that due to impaired self-awareness, some adolescents are unable to take the initiative to overcome obstacles in their growth and often take inappropriate psychological compensation means (such as online games) to meet developmental needs (73). In the adolescent growth process, good peer relationships make irreplaceable contributions to emotional cognition and social communication (74). Peer relationships can directly affect individual physical and mental growth, and loneliness is usually relatively high in poor peer relationships. When these adolescents suffer from inner pain, they are isolated and have no one to talk to because of their lack of life experience and difficulty in dealing with interpersonal relationship problems, they can feel lonely and turn to the Internet to make up for the vacancy in the inner world (75). Poor-quality interpersonal relationships aggravate loneliness and result in mobile phone obsession.

In the first half of the mediating effect of interpersonal relationship quality, loneliness was mediated by interpersonal relationship quality through the indirect influence of mobile phone anthropomorphism on mobile phone addiction. Loneliness is the distress arising from unsatisfying social connections that fail to fulfill intimacy needs (76). Positive interpersonal relationship quality and good social support can reduce individual loneliness (77). When loneliness is reduced, individuals are more likely to participate in real-life activities because of positive emotions and attitudes. Highquality interpersonal relationships can alleviate inner loneliness, thereby reducing the tendency to anthropomorphize mobile phones. When individuals perceive a low quality of interpersonal relationships and lack the value of in-depth communication, they tend to actively reduce their social interaction at a realistic level. However, their intrinsic social needs did not disappear, but instead prompted them to turn to anthropomorphic products to seek and establish social connections (16). This approach can avoid the problem of emptiness in the inner world of individuals caused by an active reduction in contact due to the perception of low-quality interpersonal relationships. Therefore, lowquality interpersonal relationships make people experience alone and are more likely to personify their phones. College students can interact with their classmates and friends online anytime and anywhere and receive online social support, which can enhance their friendships with others (45). People with high network social support also have good interpersonal relationships and low loneliness, and it is not easy to anthropomorphize their mobile phones.

## 6 Conclusions

In the study, the cluster randomized sampling approach was implemented to send out network questionnaires to college students across the country, and IBM SPSS 27.0, and PROCESS 4.1, were employed to perform descriptive statistical analysis and model tests of mediating and mediating effects, respectively. The Harman single factor test was used to assess common method bias, while Pearson’s correlation analysis was performed to investigate the connections among key variables. The complex mechanisms of loneliness, mobile phone anthropomorphism, and mobile phone addiction were revealed, providing a new view for understanding the psychological characteristics of contemporary youth and mobile phone dependence. This study found that loneliness among university students not only promoted the development of phone addiction as a direct factor but also indirectly intensified the individual trend of mobile phone addiction through this intermediary process of treating mobile phones as objects with emotional interaction ability (mobile phone anthropomorphism). This finding highlights the central role of loneliness in shaping individual behavioral patterns and the mediating effect of mobile phone anthropomorphism as a psychological coping strategy. With an increase in loneliness, college students tend to project more emotions onto their mobile phones, seeing them as tools or partners to relieve loneliness. This behavioral pattern deepens the unrealistic interaction between humans and computers, and further promotes mobile phone addiction. Simultaneously, the importance of interpersonal relationship quality as a moderating variable is verified. High quality interpersonal relationships act as a barrier that can effectively alleviate individual loneliness, thus reducing the tendency toward mobile phone anthropomorphism and the jeopardy of mobile phone addiction driven by loneliness. This suggests that building and maintaining positive supportive relationships in real-world socialization can play a significant role in preventing or reducing digitally dependent behaviors. This provides important insights for mental health education and intervention, that is, by promoting positive interactions between people, we can successfully counteract the adverse impacts of loneliness. Findings suggest that mental health interventions should prioritize fostering real-world social connections to prevent mobile phone dependency. Future programs could include: Peer mentorship programs to enhance social support networks. Digital detox initiatives that encourage students to replace screen time with in person interactions. Mindfulness based interventions to enhance self-regulation and reduce compulsive phone use.

Although this study has made progress in revealing the connection between loneliness, mobile phone anthropomorphism, and mobile phone addiction among universities, some limitations still affect the universal applicability and theoretical depth of the conclusions. As a snapshot study, this study can only provide evidence of correlation between variables but cannot directly prove the existence of causal chains. To overcome this limitation, future research could design longitudinal followup studies that directly examine how changes in loneliness affect the trajectory of mobile phone anthropomorphism and addiction through long-term observation and data collection, as well as the causal links between these processes. This study focuses on college students. Although this group had a high representation of mobile media use, the universality of the conclusions needs to be further verified. Future research should extend to groups of different ages and social backgrounds to explore the differences and commonalities of loneliness, mobile phone anthropomorphism, and mobile phone obsession among different demographic characteristics. Based on the cognitive emotion behavior model, interpersonal relationships are a complex and multidimensional concept. This study has not fully detailed how their subdivision variables interact with loneliness, mobile phone anthropomorphism, mobile phone addiction. Future studies should refine the observable variables and adopt a multidimensional analytical framework to more comprehensively understand the moderating effects of interpersonal relationships on loneliness and mobile phone addiction, which will offer a stronger scientific foundation for developing effective preventive measures and target intervention appro aches.

## Data Availability

The original contributions presented in the study are included in the article/**Supplementary Material**. Further inquiries can be directed to the corresponding author.
